# Improvement and decline of cognitive function in schizophrenia over one year: a longitudinal investigation using latent growth modelling

**DOI:** 10.1186/1471-244X-7-16

**Published:** 2007-05-09

**Authors:** Jennifer H Barnett, Tim J Croudace, Sue Jaycock, Candice Blackwell, Fiona Hynes, Barbara J Sahakian, Eileen M Joyce, Peter B Jones

**Affiliations:** 1 University of Cambridge Department of Psychiatry, Box 189 Addenbrooke's Hospital, Cambridge CB2 2QQ, UK; 2 Research & Development Department, Nottinghamshire Healthcare NHS Trust, Duncan Macmillan House, Nottingham NG3 6AA, UK; 3 Division of Neurosciences and Mental Health, Faculty of Medicine, South Kensington Campus, Imperial College, Exhibition Road, London SW7 2AZ, UK

## Abstract

**Background:**

Long-term follow-up studies of people with schizophrenia report stability of cognitive performance; less is known about any shorter-term changes in cognitive function.

**Methods:**

This longitudinal study aimed to establish whether there was stability, improvement or decline in memory and executive functions over four assessments undertaken prospectively in one year. Cognitive performance was assessed during randomized controlled trials of first- and second-generation antipsychotic medication. Analyses used a latent growth modeling approach, so that individuals who missed some testing occasions could be included and trajectories of cognitive change explored despite missing data.

**Results:**

Over the year there was significant decline in spatial recognition but no change in pattern recognition or motor speed. Improvement was seen in planning and spatial working memory tasks; this may reflect improved strategy use with practice. There were significant individual differences in the initial level of performance on all tasks but not in rate of change; the latter may have been due to sample size limitations. Age, sex, premorbid IQ and drug class allocation explained significant variation in level of performance but could not predict change. Patients randomized to first-generation drugs improved more quickly than other groups on the planning task.

**Conclusion:**

We conclude that cognitive change is present in schizophrenia but the magnitude of change is small when compared with the large differences in cognitive function that exist between patients. Analyses that retain patients who drop out of longitudinal studies, as well as those who complete testing protocols, are important to our understanding of cognition in schizophrenia.

## Background

The neurodevelopmental model of schizophrenia suggests that cognitive deficits result from aberrant early brain development [1-3], but it remains possible that further cognitive change occurs once the disease develops. Longitudinal studies generally show that cognitive deficits are stable over long periods in chronic schizophrenia although there is evidence for decline in elderly institutionalised patients [4-6]. However these studies demonstrating cognitive stability usually involve a baseline assessment and a single follow-up [7,8], so would not detect any shorter-term fluctuations in cognitive performance [9]. For example, cognitive function might fluctuate alongside day-to-day changes in symptom levels or, less dramatically, might be relatively improved during remitted phases and relatively worse during relapse.

Understanding of cognition in schizophrenia could clearly be improved by studies involving more extensive repeated assessments, and more sensitive longitudinal analyses. Given the clinical and cognitive heterogeneity in schizophrenia, a within-subject, follow-through design is necessary for individual differences to be more fully understood. Comparisons of cross-sectional samples from different points in the illness course are prone to sampling biases, so cannot answer questions about factors which may affect performance as individuals progress through the illness. However practise effects may result in artificial improvement in performance over repeated cognitive assessments in longitudinal studies. Finally, since longitudinal studies inevitably suffer from participant attrition, appropriate statistical analyses are required that make maximum use of all available data. Only then will results reflect what is known about those who drop out of the study as well as those who remain in and complete the full protocol of assessments [10,11].

These issues are of relevance to current randomised controlled trials of antipsychotic drugs. Compared with first-generation antipsychotics (FGAs), the second-generation drugs (SGAs) may ameliorate the cognitive deficit in schizophrenia [12]. However, for cognitive performance to become a consideration in clinicians' and patients' choice of drugs, robust cognitive effects must be seen when drugs are administered at a clinically appropriate dose, rather than at a dose dictated by solely by a research protocol.

This study aimed to investigate improvement and decline of cognitive function in schizophrenia in pragmatic, single-blind drug trials: the Cost Utility of the Latest Antipsychotics in Severe Schizophrenia (CUtLASS) studies. In CUtLASS, clinicians were given choice over the antipsychotic drug and dosage used for each patient. Cognitive change was characterised by a standardised neuropsychological assessment battery that was repeated four times over the course of one year. The primary aim of this report was to assess whether the group showed cognitive change over time, and the extent to which this varied between individuals. This was done using methods for modelling individual differences in the context of group mean effects: latent growth curve modelling. We predicted that there would be no significant change over one year, since the sample comprised patients with a generally chronic and stable state of illness. Our analyses were opportunistic on the collection of cognitive data on a non-randomly selected sub-sample of trial participants, although this initiative was written in to the trial protocol. Our results do not therefore inherit all the benefits of a fully-randomised design, and we cannot rule out the impact of selection factors on our results (see later Discussion).

## Methods

### Study design

Full details of the CUtLASS trials have been published elsewhere [13,14]. The present study concerns a sub-sample of patients who underwent additional cognitive investigations. Briefly, the CUtLASS studies were multi-centre randomised controlled trials, which enrolled patients from the UK National Health Service with DSM-IV schizophrenia, delusional disorder, or schizoaffective disorder.

CUtLASS I [14] compared FGA and SGA drugs in patients whose current treatment was being changed because of inefficacy or intolerability; CUtLASS II [13] compared clozapine with other SGAs in people whose medication was being changed because of poor clinical response to two or more drugs. Clinicians were free to choose the specific drug from within the randomised class (FGA or SGA), and the appropriate dosage.

In the main protocol, four clinical assessments were completed over the course of one year: at trial entry, and subsequently at 12, 26 and 52 weeks. In this cognitive sub-study, neuropsychological assessments were also completed at these four time-points. Patients were recruited for the cognitive study from the London and Nottingham centres, according to their willingness to take part in the additional assessments.

The study was approved by the Local Research Ethics committees and informed consent was obtained from all participants.

### Neuropsychological assessment

The National Adult Reading Test [NART, [15]] was administered at the first assessment to yield an estimate of premorbid IQ. Neuropsychological performance was assessed using tests from the Cambridge Neuropsychological Test Automated Battery (CANTAB) which previous studies had demonstrated are sensitive to impairments in schizophrenia [16-18]. The CANTAB motor control task was used first on each occasion, to assess current motor processing and to familiarise participants with the touch-screen system. The remaining tests were then completed in a fixed order, so that the pattern of available data would be consistent between and within patients.

#### Spatial and pattern recognition memory [19]

These tasks require participants to remember a series of five locations (spatial recognition) or 12 abstract patterns (pattern recognition) presented on the screen. After a short delay, participants choose which of a pair of locations/patterns they have seen before. This is repeated with subsequent series. The percentage of correct responses is recorded.

#### Spatial Working Memory (SWM) [19]

In this self-ordered search task, participants search boxes to find hidden tokens. Participants are told they should remember which boxes they have already looked in since the token will never appear there again. Two outcome measures are recorded: the total number of errors, and a strategy score, reflecting the number of search sequences that are begun in the same box. More efficient strategies receive lower strategy scores.

#### Stockings of Cambridge (SoC) [20]

Based on the Tower of London task [21], this test assesses planning ability. Subjects see two pictures of coloured balls in 'snooker pockets', and asked to move the balls to make one picture match the other. The minimum number of moves required is shown on the screen during the task; the number of optimal solutions, initial thinking time before the first response at the four-move level, and subsequent thinking time between responses at the four-move level are recorded.

### Variable transformations

One-sample Kolmogorov-Smirnov tests indicated that four outcome variables were not normally distributed. These were transformed to approximate normality before analysis: SoC initial and subsequent thinking times and the motor control task response times (square-root transform), and spatial working memory strategy score (squared).

### Analysis

#### Choice of analysis

Longitudinal changes in cognition are usually examined using repeated measures ANOVA but there are clear benefits in using more sophisticated techniques [22]. Since this sample contained missing data many participants would be lost from the ANOVA analysis that could be implemented in standard statistics packages such as SPSS. It was clearly preferable to use more flexible longitudinal methods that could retain partially incomplete data. This would maximise the power of the study and allow results to include information from patients who dropped out of the trials as well as those who completed the full protocol [23]. Given the known heterogeneity of cognition in schizophrenia, methods that allow consideration of individual variation in the rate of cognitive change may also be important. The chosen approach, encompassing these advantages, was to use latent growth modelling (LGM) in a structural equation modelling environment. Latent growth models (also known as random effects models) were defined and estimated using Mplus version 3.1 [24]. All models estimated in this paper could also be approached from a multilevel/linear mixed modelling perspective [23]. These models show great similarities and equivalences [24] and we would expect similar conclusions to be reached.

#### Latent growth modelling approach

For each individual, cognitive function is assumed to follow a specified function of time, plus an error component (or residual). Latent growth factors were applied in the manner of a confirmatory factor analysis to account for the temporal ordering and dependency among the repeat observations. The parameterisation of the growth factors is an attempt to smooth over the pattern of change recorded by the observed measures to estimate the continuous trajectory that might have given rise to the observations. Robust modelling of curvilinear change requires larger samples or more repeated measures. We therefore restricted our modelling to linear growth models where individual trajectories were summarised by an intercept, reflecting initial performance level, and a single slope, reflecting linear rate of change over time in each dependent variable.

An advantage of LGM is that individual variation can be considered as well as group mean effects: inter-individual differences in rate of change (slope) and initial status (intercept) were estimated and the effects of demographic and treatment variables in predicting individual differences assessed.

#### Modelling with covariates (conditional modelling)

Although initial models were performed unconditionally (without covariates), subsequent modelling was performed with covariates to improve the validity of the model for cases with missing data (because they provide information from which the model can predict the likely pattern of missing data). Since attrition or change may be related to covariates such as age, sex or drug class, inclusion of these in the model improves the likelihood that the model assumptions regarding missing data will be met. Moreover this conditional modelling approach enabled investigation of the extent to which demographic characteristics might explain individual variation in intercept and slope.

The effect of demographic characteristics and treatment allocations on individuals' level and change in performance were investigated using multivariate outcome linear regressions in conditional growth models. Modelled intercept and slope parameters were regressed on dummy variables to identify group differences. The numbers of subjects involved in the study was too small to allow the effects of individual drugs on cognition to be meaningfully examined, however binary dummy variables for drug class (FGA [1,0] SGA [0,1] and clozapine [0,0]) were included, along with age, sex, and NART-estimated premorbid IQ. Effects of these covariates on intercept or slope were considered significant if the Wald ratio (effect estimate: standard error) was greater than 1.96, according to a z-distribution.

Likelihood ratio tests were used to determine if the regression of covariates on intercept and slope terms improved the fit of the model. It was significantly improved if the deviance (-2 x the log-likelihood difference) between the conditional model, and a model where regression of the five covariates on intercept and slope terms was fixed to zero exceeded 18.3 (χ^2 ^statistic for p = 0.05 at 10 DF).

#### Modelling process

Model estimation was performed using maximum likelihood. Indices of model fit based on the returned log-likelihood value were calculated directly by the programme (Mplus Version 3.1). Maximum likelihood parameter estimates and robust standard errors returned by the ESTIMATOR = MLR option are reported; these are the default options in Mplus. Partially incomplete data were included via maximisation of the log-likelihood based on all individual data contributions. Application of the Expectation-Maximisation algorithm allows inclusion of all available data, including patients who dropped out of the trials assuming a Missing At Random mechanism [25]. Further details of the model selection process are available from the authors.

#### Missing data and dropout

In order to explore the likely validity of our modeling assumption that the cognitive data are missing at random (MAR), logistic regression was used to estimate the effects of NART IQ, age, gender and clinical state at trial entry (positive, negative and general scale scores from the Positive and Negative Syndrome Scale (PANSS, [26]) on the probability of dropping out of the study. Since this only characterises the impact of time invariant covariates on remaining in the study, we also estimated wave on wave logistic regression models to predict dropout at each cognitive assessment from cognitive score(s) at the preceding assessment.

## Results

### Patient characteristics and drug allocations

Ten subjects were enrolled into the cognitive study from London and 48 from Nottingham, comprising 17 women and 41 men in total. DSM-IV diagnoses were schizophrenia (n = 45), schizoaffective disorder (n = 9) and delusional disorder (n = 4). Further characteristics are shown in Table 1.

**Table 1:** Characteristics of the CUtLASS cognitive study sample at trial entry

	Mean (SD)	Range
Age (years)	41.9 (12.0)	22–67
Duration of illness (years)	16.5 (12.0)	1–46
PANSS Positive symptoms	16.6 (6.92)	7–37
PANSS Negative symptoms	22.0 (7.58)	7–42
PANSS General symptoms	34.5 (8.66)	7–56
Global Assessment of Function	43.3 (15.0)	17–66
NART premorbid IQ	105.6 (12.3)	87–123

Drug allocations	FGA (n)	SGA (n)
	
	Droperidol (1)	Clozapine (14)
	Haloperidol (2)	Quetiapine (4)
	Sulpiride (10)	Risperidone (4)
	Thioridazine (1)	Olanzapine (19)
	Trifluoperazine (2)	
	Zuclopenthixol (1)	

### Cognitive performance during the CUtLASS trials

Performance at trial entry was within the range of published results in schizophrenia [16-18]. There was considerable variability of cognitive performance, both between patients and within patients between testing sessions. Test performance can be seen in Table 2. The cognitive study suffered from considerable attrition, and patients were also more likely to complete the earlier than later tests within each session. Column 6 of Table 2 shows the proportion of patients for whom complete data were available.

**Table 2:** CUtLASS cognitive performance measures over one year, at 0,12, 26 and 52 weeks.

	Group performance [Mean (SD)]				Complete Data	LGM Parameters [Estimate(SE)]				Correlation
							
Cognitive Measure	0	12	26	52	N (% of sample)	Intercept	Slope	Variance of Intercept	Variance of Slope	Intercept-Slope
Motor control task (√ ms)	34.6 (5.4)	34.1 (5.8)	34.9 (6.9)	35.0 (6.9)	25 (43%)	3.45 (0.83)	0.07 (0.22)	23.0 (8.88)	0.50 (0.70)	0.15
Pattern recognition (% correct)	73.8 (16.5)	76.3 (17.0)	77.7 (17.2)	73.9 (17.1)	26 (45%)	74.7 (2.12)	0.17 (0.47)	209 (49.2)	4.22 (2.19)	-0.14
Spatial recognition (% correct)	69.8 (14.3)	67.1 (16.2)	66.7 (16.1)	63.0 (16.5)	26 (45%)	69.2 (1.79)	-1.21 (0.47)	101 (36.3)	0.70 (2.31)	0.71
SoC optimal solutions	6.22 (2.83)	6.86 (2.93)	6.93 (2.74)	7.06 (2.31)	20 (35%)	6.29 (0.37)	0.19 (0.07)	4.87 (1.54)	0.00 (0.10)	-0.65
SoC initial thinking time (√ ms)	81.7 (28.7)	76.0 (34.3)	68.1 (39.4)	67.3 (27.8)	19 (33%)	78.9 (3.91)	-2.69 (0.90)	400 (173)	19.0 (28.5)	-0.18
SoC subsequent thinking time (√ ms)	50.0 (25.7)	44.4 (27.7)	33.1 (23.2)	32.1 (24.3)	18 (31%)	48.5 (3.34)	-3.86 (0.75)	427 (162)	1.46 (9.37)	-0.52
SWM strategy score (squared)	1509 (214)	1555 (356)	1409 (324)	1304 (272)	16 (28%)	1434 (44.4)	-30.6 (9.94)	54.5 (20.3)#	3.03 (2.25)#	-0.45
SWM Total Errors	53.2 (25.8)	48.7 (24.7)	40.8 (24.7)	43.7 (30.8)	15 (26%)	53.0 (3.58)	-1.73 (0.74)	461 (130)	0.70 (4.79)	0.74

Means (SD) and repeated measures ANOVA results. Scores shown are transformed to approximate the normal distribution. # × 10^-3^

Logistic regression analyses revealed that dropout from the study was independent of NART IQ and all clinical scores at trial entry, but related to sex and age. The was a trend for men (β = 0.832; SE = 0.681, p > 0.05) and younger individuals (β = -0.066; SE 0.045, p > 0.05) to be more likely to drop out of the trial but in neither case was the effect statistically reliable, probably due to the low power of such analyses with our small sample size and the number of dropouts in each analysis.

Dropout from the second and third waves of data collection was not significantly predicted by the preceding test performance on any cognitive variables. In contrast, dropout at T4 was weakly predicted by performance at T3 on spatial working memory errors (β = -0.04; SE = 0.02; p = 0.08) and strategy (β = -0.004; SE = 0.002; p = 0.02), and by pattern recognition (β = -0.06; SE = 0.03 p = 0.04). In each case, patients who dropped out at T4 had performed better at T3 than those who stayed in. Although we cannot infer anything about whether those who dropped out did so because of the cognitive performance that they would have achieved, had they not dropped out, these logistic regression results and trends suggest to us that there is a need for, and considerable value in including missing data in the analyses under a MAR approach, since this will reduce the bias that might otherwise result from failure of the missing completely at random (MCAR) assumption, which applies to a complete case only analysis.

Repeated measures ANOVAs on this small group with complete data were severely underpowered to detect change; nonetheless significant improvement was detected in SoC subsequent thinking time (F = 4.31, DF 3,51, p < .01) and SWM strategy (F = 6.29, DF 3,34, p < .01).

Latent growth modelling was conducted as described above. Table 2 shows the estimated parameters of the LGMs for each measure (see Additional Files 1 and 2 for further details). For every variable, the intercept (initial performance) differed reliably from zero. In all but two cognitive variables (pattern recognition and motor latency), the group mean slope also differed reliably from zero, representing a significant improvement in cognitive performance over time, with the exception of spatial recognition memory where a significant decline in performance was seen.

In all models there was significant within-group variation in intercept, but contrary to expectations, there was no reliable within-group variation in slope. For all measures, the magnitude of the slope was small relative to the variance in intercept, implying that mean change is small, compared with the individual differences in absolute performance level.

Significant amounts of variance in intercept and slope terms remained unexplained. The modelling process was therefore repeated with covariates, to determine the extent to which age, sex, NART-estimated IQ and treatment allocation explained the variance in intercept and slope, and any additional variance.

### Effects of covariates including drug class on cognition

For each cognitive measure, age, sex, NART and drug class were assessed as predictors of individual differences in intercept and slope. These results are shown in Table 3; the estimate and standard error of the effects of each covariate on intercept and slope are shown first, followed by the percentage of variance explained (r^2^) for continuous, and effect size (d) for binary covariates. Effects shown in bold are statistically significant (ratio of estimate: standard error > 1.96). The direction of effects on intercept and slope are shown in the bottom two rows, describing variables that predict better performance at trial entry and faster improvement over time.

**Table 3:** Multivariate regression of covariates on latent growth models of cognitive measures in CUtLASS.

	Pattern Recognition		Spatial Recognition		SWM Errors		SWM Strategy		SoC Solutions		SoC Initial Thinking		SoC Subsequent Thinking		Motor Latency	
	Est (SE)	r^2^/d	Est (SE)	R^2^/d	Est (SE)	r^2^/d	Est (SE)	r^2^/d	Est (SE)	r^2^/d	Est (SE)	r^2^/d	Est (SE)	r^2^/d	Est (SE)	r^2^/d
Intercept:																
NART-IQ	**0.47 (0.19)**	**0.31**	0.21 (0.18)	0.18	**-1.08 (0.29)**	**0.46**	-0.08 (0.05)	0.33	**0.14 (0.02)**	**0.60**	-0.13 (0.08)	0.25	**-0.92 (0.29)**	**0.41**	-0.13 (0.08)	0.25
Age	**-0.35 (0.17)**	**0.29**	**-0.31 (0.15)**	**0.32**	**1.03 (0.25)**	**0.53**	0.01 (0.04)	0.03	**-0.13 (0.02)**	**0.63**	**0.14 (0.07)**	**0.33**	**1.30 (0.22)**	**0.69**	**0.14 (0.07)**	**0.33**
Sex	**10.6 (4.04)**	**0.73**	**-7.86 (3.61)**	**0.69**	**14.8 (6.43)**	**0.64**	0.85 (1.06)	0.35	**-1.36 (0.57)**	**0.55**	3.04 (1.71)	0.60	8.59 (5.64)	0.38	3.04 (1.71)	0.60
FGA	5.91 (5.02)	0.41	-2.70 (4.50)	0.24	-2.55 (7.87)	0.11	-1.02 (1.25)	0.42	-0.93 (0.70)	0.38	2.67 (2.08)	0.53	4.29 (7.16)	0.19	2.67 (2.08)	0.53
SGA	2.17 (4.60)	0.15	-1.15 (4.13)	0.10	12.55 (6.81)	0.54	-0.32 (1.02)	0.13	**-1.46 (0.61)**	**0.59**	2.06 (1.92)	0.41	**21.8 (6.24)**	**0.98**	2.06 (1.92)	0.41

Slope:																
NART-IQ	0.02 (0.05)	0.09	0.09 (0.05)	0.59	-0.05 (0.08)	0.21	-0.01 (0.01)	0.19	-0.01 (0.01)	0.31	0.03 (0.02)	0.40	0.07 (0.09)	0.21	0.03 (0.02)	0.40
Age	-0.08 (0.04)	0.46	-0.01 (0.04)	0.09	-0.02 (0.07)	0.10	**0.02 (0.01)**	**0.44**	0.00 (0.01)	0.08	**0.04 (0.02)**	**0.55**	**-0.16 (0.07)**	**0.55**	**0.04 (0.02)**	**0.55**
Sex	1.35 (0.87)	0.68	-1.51 (0.84)	1.05	**-3.76 (1.66)**	**1.72**	0.01 (0.04)	0.00	**0.42 (0.13)**	**0.55**	-0.27 (0.45)	0.32	-1.52 (1.51)	0.44	-0.27 (0.45)	0.32
FGA	-0.27 (1.12)	0.14	-0.46 (1.08)	0.32	-1.29 (2.05)	0.63	-0.22 (0.25)	0.50	**0.33 (0.17)**	**0.96**	-0.47 (0.55)	0.56	-3.53 (1.90)	1.03	-0.47 (0.55)	0.56
SGA	0.77 (1.01)	0.39	-1.02 (0.99)	0.36	-1.40 (1.70)	0.64	0.04 (0.20)	0.08	0.12 (0.15)	0.36	-0.01 (0.52)	0.01	-3.21 (1.67)	0.93	-0.01 (0.52)	0.01

Better at trial entry	MEN, YOUNGER HIGHER IQ		MEN, YOUNGER		WOMEN, YOUNGER HIGHER IQ		-		MEN, YOUNGER HIGHER IQ, SGA,		YOUNGER		YOUNGER, HIGHER IQ		YOUNGER	

Faster to improve	-		-		MEN		YOUNGER		WOMEN, FGA		OLDER		OLDER		YOUNGER	

Covariates are: NART-predicted IQ, age, sex, drug class [FGA,SGA,clozapine]. Regression of covariates on 'best-fit' latent growth models. **Bold **= Wald Ratio (Estimate: Standard Error) reliable at p < 0.05 (i.e. significant effect on growth factor). Effect sizes: **r**^2 ^given for continuous covariates (age, NART); Cohen's d for categorical ones (sex, FGA, SGA)

**Figure 1:**
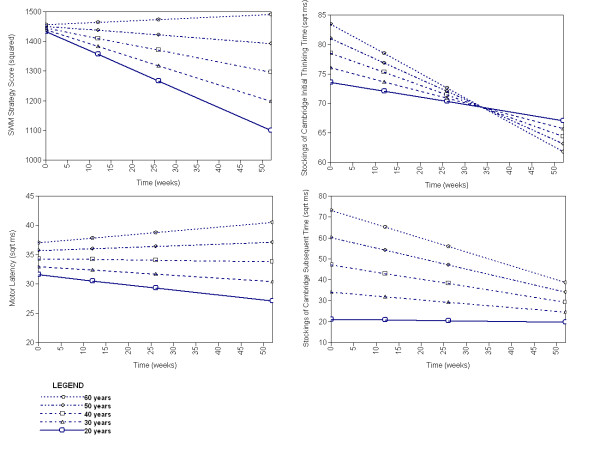
**Modelled effects of age at trial entry on cognitive performance across one year in schizophrenia**. Faster/better performance at lower part of graph.

**Figure 2:**
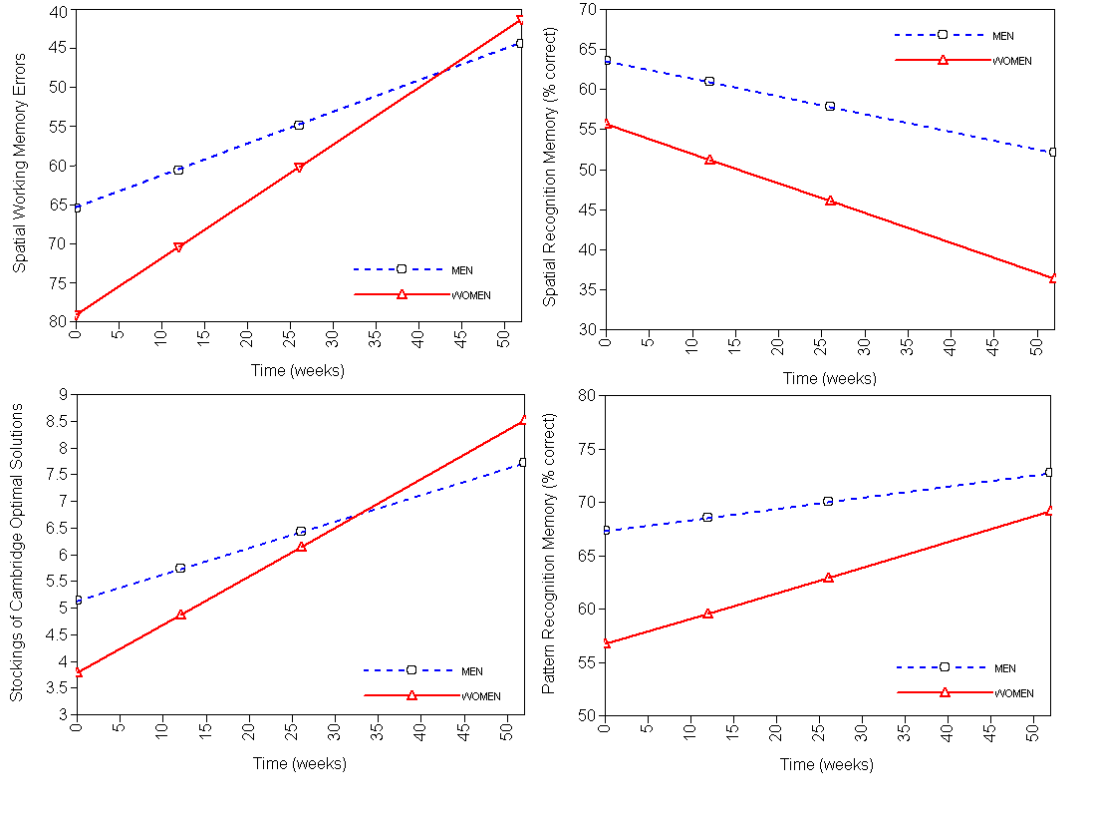
**Modelled effects of sex on initial performance and rate of change of cognition over one year in schizophrenia**. Better performance at upper part of graph.

**Figure 3:**
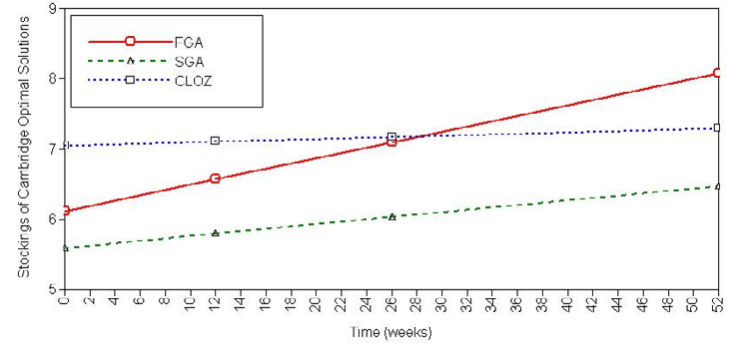
**Effects of drug class allocation (first-generation antipsychotic; second-generation antipsychotic, clozapine) on Stockings of Cambridge Optimal Solutions over one year in schizophrenia**. Significant differences exist between SGA and other classes at trial entry; rate of change over time is greater in FGA than other classes. Better performance at upper part of graph.

Sex, age, or NART-IQ significantly predicted the intercepts of all variables except SWM strategy. Allocation to the SGA group affected the intercepts of SoC optimal solutions and subsequent thinking times. Predictors of cognitive change were less common, and were generally limited to age (see Figure 1) and sex (see Figure 2). Treatment allocation affected only one measure, SoC optimal solutions, where those allocated to a FGA improved more quickly than those allocated to other drugs (see Figure 3).

Likelihood ratio tests showed that covariates significantly improved the fit of all models except SoC initial thinking time (deviance = 7.46, required χ^2 ^= 18.3).

## Discussion

Conventional analysis using repeated measures ANOVA revealed significant improvement over time in two variables (SoC subsequent thinking time and SWM strategy) but was limited by the exclusion of up to 74% of cases due to missing data. This level of attrition resulted in a loss of power to just 17% for a cognitive change of 0.5 standard deviations at an alpha level of 0.05. Latent growth modelling using maximum likelihood methods allowed inclusion of all available data and consequently increased power and representativeness, although our sample was still somewhat selected. LGM revealed significant improvement over time in all cognitive variables except pattern recognition and motor latency, and spatial recognition memory, which declined over time.

Traditional statistical techniques that only allow inclusion of complete data cases may mask individual variations in cognitive change, by reducing the power to detect reliable individual or group differences, or limit generalisability, due to exclusion of a non-random subset of participating patients. Here, statistical modelling in a LGM framework using maximum likelihood estimation enabled the performance of those who dropped out of the study to be included in analyses, and analysed alongside study completers. Longitudinal data of this kind benefit from careful analysis, especially in small samples or where missing data occurs.

Although unconditional LGM is an improvement over techniques that exclude data with missing values, these models must still be interpreted with caution since for all cognitive measures, significant amounts of variance remained unexplained. Conditional modelling, by improving the likely validity of the missing data assumptions improved the fit of all models except one, and should also have improved the external validity of the study.

There was no clear pattern of factors that predicted cognitive change throughout the study. In general, being young, male, and of a higher NART IQ predicted better initial performance. The effect of age on rate of change varied: in some cases older patients improved less quickly, in others more quickly, perhaps where younger patients had already reached a performance limit (e.g. SoC subsequent thinking time, Figure 1). Similarly, although women often started with poorer performance they tended to improve more quickly (Figure 2).

For two variables, (SoC optimal solutions and subsequent thinking time) patients on second-generation drugs were cognitively worse at trial entry. This probably reflects the randomisation process of the two trials: half of those allocated SGAs and all of those allocated to clozapine were from the CUtLASS II trial of patients where at least two previous antipsychotics had failed to control their symptoms, while this was not the case for any patient randomised onto FGAs. Patients allocated to SGAs may therefore have been, on average, more symptomatic, or ill for longer than those randomised to FGAs.

Treatment allocation affected the rate of change on one cognitive measure, with patients randomised to the FGA group showing improved SoC performance more quickly than patients allocated SGA drugs, including clozapine. Although contrary to the general belief that SGAs improve cognition more than FGAs [12,27] this is interesting since CUtLASS I [14] found no evidence for the presumed benefits of SGAs over FGAs in treatment efficacy. Overall, drug class had little impact on cognition in this study, although this may largely reflect a lack of power. Drug-by-drug analyses, which may have been informative, were not possible due to the small sample.

Consistent improvement was seen throughout the year in the planning and working memory tasks, but not in spatial or pattern recognition. One interpretation of this is that patients benefited from practise only on tasks that have some strategic component. This is interesting given previous reports of a relative inability of patients with schizophrenia to benefit from strategy training [28,29]. Another possibility is that the general clinical improvement seen in the CUtLASS trials [13,14] affected memory less than executive functions, which might be more sensitive to changes in dopaminergic function. Either way, given that a minimum of 12 weeks existed between assessments, the significant improvements seen over time in the planning and working memory tasks are noteworthy.

There was no evidence of improvement with practise for pattern recognition, while decline was seen in spatial recognition. In contrast, healthy volunteers improve on pattern recognition at a four-week retest while their performance on spatial recognition remains stable over time [30]. This could reflect structural changes such as the grey matter reductions found in first-episode psychosis [31-33], or alternatively, an acceleration of the normal age-related worsening of memory. Some authors have argued for accelerated cognitive decline in elderly schizophrenia patients but this has usually been restricted to executive functioning [34]. Moreover, recent research has found no significant difference in age-related cognitive decline in adults with schizophrenia compared with healthy controls [35].

These findings may be limited by the drug trial context. The CUtLASS trials had the advantage of randomised drug class allocation, avoiding the problem in naturalistic studies where certain drugs may be prescribed for particular groups. However, they were not designed with cognition as the primary endpoint, so our results regarding the randomised groups should be treated with caution: randomisation may not be complete for this selected cognitive sub-study. Nonetheless, since antipsychotic medication is usually ignored in naturalistic studies of cognition, and polypharmacy and changes of drug are not unusual in the treatment of schizophrenia, [36,37] this group may not be particularly unrepresentative of chronic schizophrenia.

In summary, both improvements and decline in cognition may occur in established schizophrenia over one year. However, cognitive change in established schizophrenia is small when compared with the large differences in cognitive function that exist between patients.

## Conclusion

Both improvements and decline in cognition may occur in established schizophrenia over one year. However, cognitive change in established schizophrenia is small when compared with the large differences in cognitive function that exist between patients.

## Competing interests

The author(s) declare that they have no competing interests.

## Authors' contributions

All authors contributed to the drafting and revision of the manuscript. PBJ, BJS and EMJ conceived and planned the study; SJ, CB and FH acquired the data; JHB, TJC and PBJ completed the statistical analysis and data interpretation.

## Pre-publication history

The pre-publication history for this paper can be accessed here:



## Supplementary Material

Additional File 1 Model fit indices, residual variance and variance explained by the model for unconditional latent growth models of cognitive performance in the CUtLASS trials. Click here for file

Additional File 2 Model fit indices, residual variance and variance explained by the model for conditional latent growth models of cognitive performance in the CUtLASS trials. Click here for file
